# Effect of progestin on thyroid function in female Wistar rats

**DOI:** 10.3389/fendo.2024.1362774

**Published:** 2024-06-05

**Authors:** Honglei Xie, Tingting Qian, Lanchun Liu, Rong Sun, Wenjing Che, Meng Zhao, Xin Hou, Haowen Pan, Yue Su, Jia Li, Xiaoqiu Dong, Peng Liu

**Affiliations:** ^1^ Center for Endemic Disease Control, Chinese Center for Disease Control and Prevention, Harbin Medical University, Harbin, China; ^2^ Endemic Disease Control Section, Yun nan Institute of Endemic Diseases Control and Prevention, Dali, China; ^3^ Ultrasound Department, The Fourth Affiliated Hospital of Harbin Medical University, Harbin, China; ^4^ Key Lab of Etiology and Epidemiology, Education Bureau of Heilongjiang Province and Ministry of Health, Heilongjiang Provincial Key Laboratory of Trace Elements and Human Health, Harbin, China

**Keywords:** progestin, female, Wistar rats, thyroid, function

## Abstract

**Introduction:**

To characterize the influence of female-specific hormones on women’s thyroid function, the study investigated the influence of extra progestin from oral contraceptives on inducing thyroid dysfunction.

**Methods:**

Sixty female Wistar rats were divided into six groups based on levonorgestrel or desogestrel administration as the main active agents: control, low (0.0039 mg*20-fold), medium (0.0039 mg*100-fold), high (0.0318 mg*100-fold) levonorgestrel (pure product); and low (0.0083 mg*20-fold) and high (0.0083 mg*100-fold) desogestrel (pure product). Progestin was administered by gavage every 4 days for 1 month. Statistical analysis was performed using one-way analysis of variance and the Kruskal–Wallis test.

**Results:**

Following levonorgestrel gavage, serum free T_4_ and thyroidstimulating hormone levels were significantly lower in the experimental group than that in the control group (*p*=0.013 and 0.043). After desogestrel gavage, the serum free T_4_ and free T_3_ levels were lower in the experimental group than that in the control group (*p*=0.019 and 0.030). Thyroid hormone antibody concentrations were lower in rats administered levonorgestrel and desogestrel than that in control rats. Moreover, exposure to progestin upregulated the expression of the thyroid-stimulating hormone receptor and sodium iodide symporter in thyroid.

**Discussion:**

Progestin stimulation enhanced the proliferation of follicular epithelial cells in rat thyroid tissues. Progestin exposure could cause thyroid dysfunction by upregulating the transcription of thyroid-stimulating hormone receptor and sodium iodide symporter in thyroid, thus inducing pathomorphological changes in rats’ thyroid.

## 1 Introduction

The thyroid gland is the largest endocrine gland in the body and is an important organ of the endocrine system (1). Its main physiological function is to synthesize thyroid hormones (THs) and regulate body metabolism. THs are closely related to body development and growth (2, 3), and are important for the normal function of almost all tissues. THs exert biological effects by binding to TH receptors (THRs) (4). The THR subtypes THRα and THRβ are expressed as different genes (5). Thyroid function is a collective indicator of TH levels, which can be monitored by measuring thyroid-stimulating hormone (TSH), free T_3_ (FT_3_), free T_4_ (FT_4_), anti-thyroid peroxidase antibody (TPOAb), and anti-thyroglobulin antibody (TgAb). FT_3_ and FT_4_ are regulated via negative feedback by the TSH and thyrotropin-releasing hormone to maintain normal levels in the blood (6, 7).

In recent years, the incidence of thyroid disease has increased sharply. The incidence of thyroid nodules has increased from nearly 10% to 20% (8); the prevalence of thyroid cancer has increased rapidly by approximately 20% annually (9). Additionally, the incidence of thyroid disease in women is higher than that in men (10, 11). In 2021, the Tumors Registry Association reported a global incidence of thyroid cancer of 3.2/10000 in women and 1.2/10000 in men (12). In Korea, the female-to-male incidence ratio of thyroid cancer is nearly 5:1, with thyroid cancer being the most common type of cancer in women (13). Moreover, epidemiological surveys reported a 4–5-fold higher prevalence of thyroid nodules in women than in men, which increased with age (14). The incidence of thyroid disease was comparable between women in menopause and men aged >40 years (15). Women have a higher risk of developing thyroid disease than do men. Sex-specific factors may interfere with thyroid function, leading to a higher incidence of thyroid disease in women.

An increasing number of women of childbearing age delay pregnancy, leading to an increased use of contraception methods. Contraceptive drugs are generally oral contraceptives (OCs) and are typically administered to women. Since the first hormonal contraceptive pill was introduced in 1960, “the pill” has become the most widely used reversible hormone contraceptive method worldwide (16). Recently, the market for OCs has expanded as individuals are increasingly interested in accurate contraception, healthy fertility, and increased awareness of contraceptives. According to the National Bureau of Statistics, OC sales in China reached 1.96 billion in 2019, an 8.8% increase from 2018. OCs are synthesized mainly using low doses of estrogen and progesterone (17). Intervention experiments including female athletes taking OCs to adjust their menstrual cycle showed that their thyroid function and endocrine system were greatly impacted (18). Thus, considering the close association between women and progestin, a primary component of OCs, progestin might affect thyroid function. Significant changes in FT_4_ and TSH levels after the use of OCs have been reported (19). Abnormal TH metabolism usually affects thyroid antibodies. For instance, high iodine can cause autoimmune thyroiditis (20), and an increasing prevalence of autoimmune thyroiditis typically accompanies hypothyroidism (21).

Different types of contraceptive pills [including short- and long-acting contraceptives and the emergency contraception pill (ECP)] have different formulas by adjusting the two medicine components (22). The contraceptive principle is multifaceted and varies according to the composition, formulation, dose, and usage. For example, the combination of estrogen and progesterone is mainly used to inhibit ovulation, low doses of progesterone impede fertilization, and high doses of progestin inhibit implantation.

As the basic active agent of hormonal contraception (23), the progesterone component of OCs provides the main contraceptive mechanism. According to their extraction, processing methods, and configurations, progestin drugs are categorized as natural or synthetic. Natural progestin is derived from plant extracts, while synthetic progestin is mostly derived from progesterone or testosterone derivatives. Common synthetic progestins include levonorgestrel (LNG), desogestrel (DSG), and drospirenone. In addition to effective contraception, combined OCs provide other important health benefits, including improving menstrual bleeding and related symptoms and reducing the risk of iron-deficiency anemia (16).

Considering the impact of progestin on women, this study assessed the effect of increasing progestin exposure on endocrine levels within the body. Specifically, we evaluated whether such exposure could induce disruptions in endogenous hormone balance and subsequently contribute to thyroid disorder onset.

## 2 Materials and methods

### 2.1 Ethics statement

All procedures on animals followed the Guide for the Care and Use of Laboratory Animals published by the Ministry of Health of the People’s Republic of China. The animal study was reviewed and approved by the Ethics Committee of Harbin Medical University (approval number: hrbmu202108). The study is reported in accordance with the ARRIVE guidelines.

### 2.2 Hormone administration

To ensure comparability to humans, hormone treatments on animals were designed to mimic clinically used human OC prescriptions. Based on the equivalent dose ratio between experimental animals and humans, the clinical dose of progestin in adult females was converted into the amount needed for one estrus cycle in sexually mature rats; subsequently, the experimental dose was increased according to a certain ratio. The estrus cycle refers to the reproductive cycle of female rats. Using a human body weight of 60 kg as a standard, the conversion factor from humans to rats was 5.98.According to the equivalent dose conversion between animals and humans in drug experiments, Db=Da*Rab (Db and Da are the standard body weight doses, 0.2 kg for rats and 60 kg for humans; Rab is the mg/kg dose conversion factor of standard animal a to b, which is 5.98 for human to rat). The proposed clinical dose was based on the same type of drug (24); generally, 50–100 fold and 10–20 fold doses were used as the high and low experimental doses, respectively.

Yutin (Beijing Zizhu Pharmaceutical Co., Ltd., Beijing China), an ECP, contains levonorgestrel (LNG). In the clinical setting, the prescribed monthly dosage of LNG is 1.5 mg, and the equivalent dose was 0.0039 mg/kg in rats. Yakutin (Beijing Resources Zizhu Pharmaceutical Co., Ltd, Beijing China) is a long-acting contraceptive that includes LNG as its progestin component. Its recommended monthly clinical dosage is 12 mg, with an equivalent dose in rats of 0.0318 mg/kg. Meanwhile, Mafron (N.V. Organon) is a short-acting contraceptive that incorporates desogestrel (DSG) as its progestin component. The prescribed monthly dosage for Mafron is 3.15 mg, with an equivalent dose in rats of 0.0083 mg/kg. The low and medium-dose groups within the LNG group were established based on the dosage of ECPs, while the high-dose groups were determined based on the dosage of long-acting contraceptives. Similarly, the low and high-dose groups within the DSG group were designed according to the dosage of short-acting contraceptives. The groups of rats and specific gavage doses are shown in **Supplementary Table S1**.

### 2.3 Rat model of long-term progestin exposure

Healthy 5-week-old female Wistar rats (without specific pathogens), weighing 120–150 g (Beijing Vital River Laboratory Animal Technology Co., Ltd., Beijing China) were housed in natural light at room temperature (22 ± 3 °C), and fed with water (water iodine content of 50 μg/L). After 1 week of acclimatization, they were randomly divided into one control group and five experimental groups (control; low, medium, and high LNG groups; and low and high DSG groups), with 10 rats in each group. After 1 week of acclimatization feeding, daily vaginal smears were collected to determine the estrus cycle [the regular estrus cycle in female sexually mature rats usually lasts 4–5 days and is generally divided into four stages: proestrus, estrus, metestrus, and diestrus (24, 25)]. Progestin was purchased in powder form and dissolved in peanut oil; subsequently, gavage every four days was performed after two vaginal smears had been established.

### 2.4 Collection of urine, blood, and tissue samples and detection

After the 30-day progestin treatment, urine was collected and the rats were euthanized. The rats were anesthetized by intraperitoneal injection of 10% chloral hydrate and blood was collected from the abdominal aorta. Blood samples were centrifuged and serum was separated and frozen at -80°C in an ultra-low temperature refrigerator until FT_3_, FT_4_, TSH, TPOAb, TgAb, and progesterone assays were performed. Tissues, including hearts, livers, and kidneys, were collected immediately after euthanization, and all tissue samples were snap-frozen in liquid nitrogen and stored at −80°C. Thyroid glands were carefully dissected and routinely processed for light microscopy examination, including 4% paraformaldehyde fixation, wax embedding, and preparation of 4 μm-thick sections. Thyroid tissue sections were stained with hematoxylin and eosin. Three regions each with 20 intact thyroid follicles were selected from the thyroid lobes. The numbers of thyroid cells (the number of visible nuclei) were then measured per follicular section (26).

The urinary iodine concentration (UIC) was determined using As^3+^-Ce^4+^ catalytic spectrophotometry. The content of iodine in urine was determined using the “Determination of iodine in urine - Part 1- Arsenic and cerium catalytic Spectrophotometry” (WS/T 1071-2016). The basic principle is to use the catalytic effect of iodine on the arsenic and cerium REDOX reaction under acidic conditions, the yellow Ce^4+^ in the reaction is reduced to colorless Ce^3+^, the higher the iodine content, the faster the reaction speed, the less Ce^4+^ remaining, the lighter the color. The absorbance value of the remaining Ce^4+^ in the system was determined at 420 nm by controlling the reaction temperature and time. The iodine content was calculated by using the linear relationship between the mass concentration of iodine and the absorbance value. The serum concentrations of thyroid hormones/antibodies and progesterone were measured using commercially available rat FT_3_, FT_4_, TSH, TPOAb, TgAb, and progesterone ELISA kits (Andy Gene, Shanghai, China), following the manufacturer’s instructions. ELISA kits with the same lot number and expiration date were used for each evaluated hormone.

Reverse-transcription quantitative polymerase chain reaction (RT-qPCR) analysis was performed to detect THRα and THRβ messenger RNA (mRNA) levels in the heart, liver, and kidneys, as well as those of thyroid-stimulating hormone receptor (TSHR) and sodium iodide symporter (NIS) mRNA in the thyroid of the rats. The excised fresh tissues were used for RNA extraction using Trizol. Reverse transcription was performed on total RNA using PrimeScript RT reagent kits with gDNA Eraser (Takara Bio, Tokyo, Japan) to synthesize cDNA. The final volume of the PCR reaction system was 10 μL (1 μL cDNA sample, 5 μL SYBR qPCR mix, 0.5 μL each primer, and 3 μL RNase-free water). GAPDH acted as a control for TSHR, NIS, THRα, and THRβ, and the PCR primers for TSHR, NIS, THRα, and THRβ were obtained from Ruibiotech Inc. (Beijing Rui Bo Xing Ke Biotech Co., Beijing China). The specific sequence of primers used is as follows:

GAPDH : ACTCCCATTCTTCCACCTTTG/CCCTGTTGCTGTAGCCATATT;TSHR: GTTCCTGAGAATCGTGGTATGG/TGGCACGGTCAGTTTGTAG;NIS : CAGAACCATTCCCGGATCAA/CCCACTTAGAAAGTCCAGAAGG;TRα:GCTGTTAATGTCAACAGACCGCT/CGATCATGCGGAGGTCAGTC;TRβ:CGGAAGGTGGCAAGGTTGAT/TTCACAGGGCAGCTCACAA.

The qPCR reaction was pre-denatured at 95°C for 10 min, 95°C for 15 s, and 60°C for 1 min for 40 cycles. All experiments were repeated three times in each group, and 2^-ΔΔCt^ method was used to quantify TSHR, NIS, THRα, and THRβ mRNA levels.

### 2.5 Statistical analysis

An Excel database was established, and SPSS version 22 (Polar Engineering and Service Consulting, Alaska, US) was used for statistical analysis. Normal and skewed distribution measurement data are expressed as means (standard deviation) and median (upper and lower quartiles) (M[IQR]), respectively. One-way ANOVA was used to determine serum hormone levels; *post-hoc* comparisons of the means were performed using the SNK-q test. Since the UIC was not distributed normally, the groups were compared using the Kruskal–Wallis test. All tests were two-sided, and statistical significance was set at *p*<0.05.

## 3 Results

This study tested serum progesterone level, UIC, serum TH levels (including FT_3_, FT_4_, TSH, TPOAb, and TgAb), mRNA expression of TSHR and NIS in the thyroid, and the THRα and THRβ mRNA expression in the tissues (hearts, livers, and kidneys) in female Wistar rats. Female sexually mature Wistar rats were chosen as the experimental animals and were gavaged to avoid the estrus period considering the method of OC consumption, after two consecutive regular estrus cycles. By gavaging progestin to female Wistar rats; measuring serum TH levels, the expression of TSHR and NIS mRNA in the thyroid, and the expression of THRα and THRβ mRNA in the tissues; and performing H&E staining on thyroid tissues, the effects of progestin on the thyroid glands of the rats were analyzed.

### 3.1 Serum progesterone in rats

**Figure 1:**
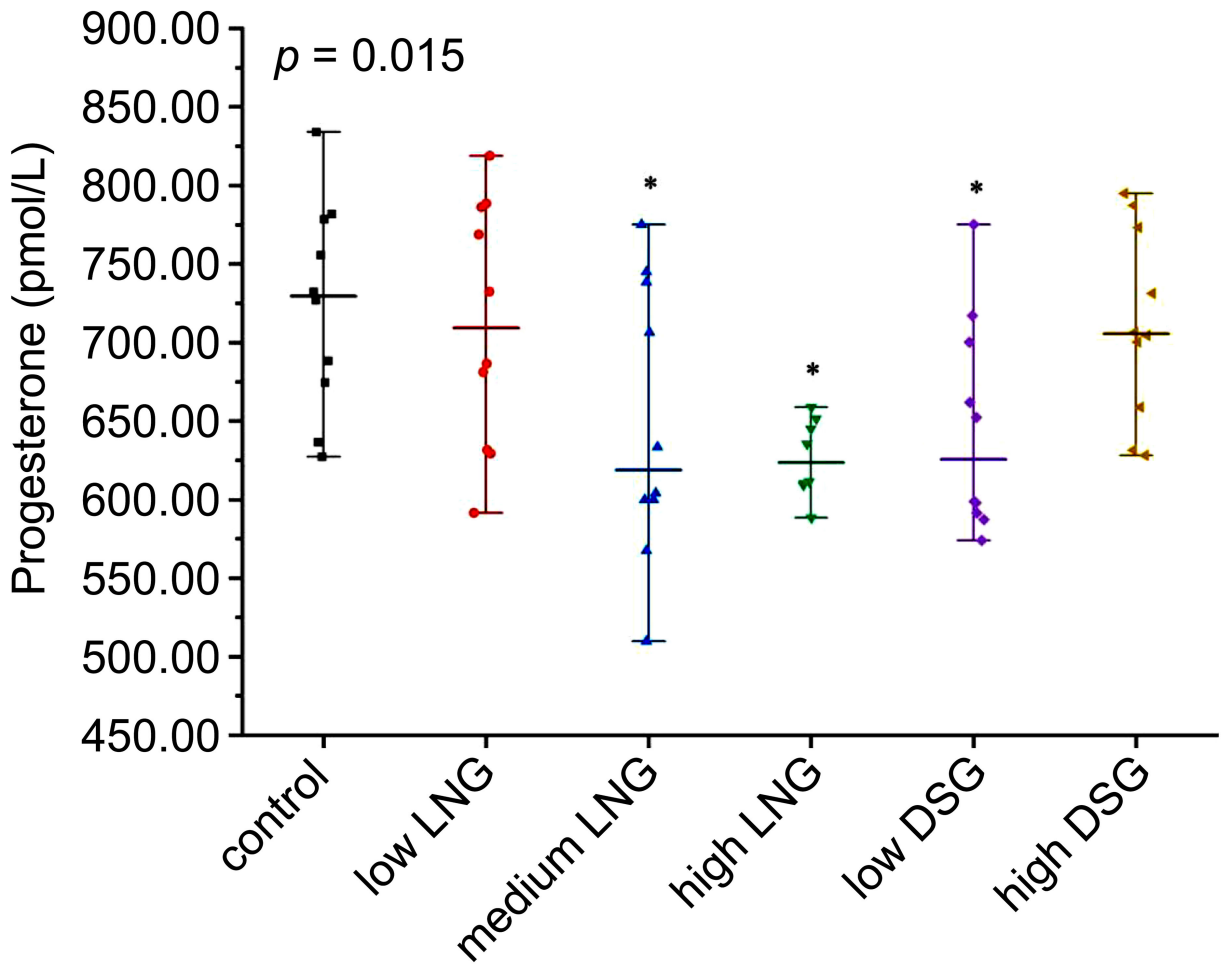
Level of serum progesterone in the rats. LNG, levonorgestrel; DSG, desogestrel; *p< 0.05 was considered statistically significant, compared with the control group.

As shown in **Figure 1**, serum progesterone levels differed significantly between the control and experimental groups (*p*=0.015). After *post-hoc* examination, the serum progesterone levels in the medium and high LNG and low DSG groups were significantly lower than those in the control group.

### 3.2 Body weights, thyroid weight, relative weight of thyroid, and UIC following exposure to progestin

After 30-day progestin treatment, the variation trends in the body weight and thyroid organ coefficient of the rats were similar. Progestin did not result in a significant change in body weight or relative weight of the thyroid (*p*=0.488 and *p*=0.363, respectively). The UIC of all experimental groups, except for the low DSG group, was higher than that in the control group. However, the difference was not statistically significant (*p*=0.597) (**Table 1**).

**Table 1:** Effect of progestin on the body weight and the organ coefficient of the thyroid in rats.

Group	Body Weight (g)	Organ Coefficient of Thyroid (%)	UIC(μg/L)
Control	273.92 ± 16.18	0.086 ± 0.015	64.13(45.55,136.64)
Low LNG	272.40 ± 14.94	0.072 ± 0.027	75.61(52.14,94.92)
Medium LNG	262.13 ± 16.31	0.072 ± 0.013	71.03(62.59,93.12)
High LNG	268.67 ± 21.31	0.065 ± 0.017	135.44(63.63,184.76)
Low DSG	267.88 ± 10.27	0.068 ± 0.006	63.95(35.05,118.72)
High DSG	274.89 ± 14.10	0.070 ± 0.016	77.30(35.09,121.42)
*P*	0.488	0.363	0.597

Results are presented as $\bar{x}$ ± s for normal distribution data and median (25^th^, 75^th^) for skewed distribution data. LNG, levonorgestrel; DSG, desogestrel; UIC, urinary iodine concentration. p< 0.05 was considered statistically significant.

### 3.3 Serum TH levels under progestin exposure

After the 30-day LNG gavage, serum FT_4_ and TSH levels differed significantly between the control and experimental groups (*p*=0.013 and *p*=0.044) (**Table 2**). After *post-hoc* examination, the serum TSH level in the high LNG group was significantly lower than that in the control group. Significant differences in the serum levels of thyroid hormone antibodies, namely TPOAb and TgAb, were observed between the control and experimental groups (*p*=0.010 and *p*=0.007). After *post-hoc* examination, the serum TPOAb levels in the high LNG group were significantly lower than those in the control group, and the serum TgAb levels in the medium and high LNG groups were significantly lower than those in the control group. The serum FT_3_ levels did not differ significantly between the control group and any of the progestin-treated groups (*p*=0.566).

**Table 2:** Effect of LNG on the thyroid function of rats.

Group	FT_3_ (pmol/L)	FT_4_ (pmol/L)	TSH (pg/ml)	TPOAb (ng/L)	TgAb (μg/L)
Control	0.80 (0.75, 0.86)	6.50 (5.78, 6.68)	39.65(35.45,46.15)	726.68(700.61,782.93)	8.60 (7.94, 8.98)
Low LNG	0.85 (0.74, 0.85)	5.75 (5.70, 6.31)	39.47(36.38,45.41)	738.23(696.55,779.26)	7.50 (7.08, 8.36)
Medium LNG	0.78 (0.74, 0.81)	5.55 (5.06, 6.11)	35.95(31.13,42.16)	679.18(661.91,713.74)	7.45 (7.00, 8.26)^a^
High LNG	0.75 (0.73, 0.90)	5.55 (4.98, 6.05)^b^	34.18(30.44,37.14)^ab^	656.45(629.45,707.55)^ab^	7.30 (7.13, 7.70)^a^
P	KW-H=2.029 p=0.566	KW-H=10.729 p=0.013	KW-H=8.110 p=0.044	KW-H=11.453 p=0.010	KW-H=12.179 p=0.007

Results are presented as $\bar{x}$ ± s for normal distribution data and median (25^th^, 75^th^) for skewed distribution data. LNG, levonorgestrel. FT_3_, free T_3_; FT_4_, free T_4_; TSH, thyroid stimulating hormone; TPOAb, anti-thyroid peroxidase antibodies; TgAb, anti-thyroglobulin antibodies. p< 0.05 was considered statistically significant. ^a^ p<0.05 vs. Control; ^b^ p<0.05 vs. Low LNG.

After the 30-day DSG gavage, serum FT_3_ and FT_4_ levels differed significantly between the control and experimental groups (*p*=0.019 and *p*=0.027) (**Table 3**). After *post-hoc* examination, the serum FT_4_ level of the low DSG group was significantly lower than that in the control group. Meanwhile, no significant differences were observed in serum TSH level (*p*=0.797). Similar to LNG, the serum TPOAb and TgAb levels in the experimental groups were lower than that in the control group after DSG gavage (*p=*0.003 and *p=*0.001).

**Table 3:** Effect of DSG on the thyroid function of rats.

Group	FT_3_ (pmol/L)	FT_4_ (pmol/L)	TSH (pg/ml)	TPOAb (ng/L)	TgAb (μg/L)
Control	0.80 (0.75,0.86)	6.50 (5.78, 6.68)	39.65 (35.45,46.15)	726.68 (700.61,782.93)	8.60 (7.94, 8.98)
Low DSG	0.80 (0.75,0.80)	5.78 (5.43, 6.15)^a^	38.45 (36.68,40.55)	613.83 (598.46,652.04)^a^	6.95 (6.78, 7.73)^a^
High DSG	0.85 (0.84,0.91)^b^	6.50 (6.01, 6.91)^b^	39.80 (34.09,42.34)	663.20 (595.35,723.71)	8.00 (7.50, 8.37)
P	KW-H=7.889 p=0.019	KW-H=7.221 p=0.027	KW-H=0.454 p=0.797	KW-H=11.930 p=0.003	KW-H=14.846 p=0.001

Results are presented as $\bar{x}$ ± s for normal distribution data and median (25^th^, 75^th^) for skewed distribution data. DSG, desogestrel; FT_3_, free T_3_; FT_4_, free T_4_; TSH, thyroid stimulating hormone; TPOAb, anti-thyroid peroxidase antibodies; TgAb, anti-thyroglobulin antibodies. p< 0.05 was considered statistically significant. ^a^ p<0.05 vs. Control, ^b^ p<0.05 vs. low DSG.

### 3.4 Transcription of target genes involved in the hypothalamic-pituitary-thyroid axis following progestin exposure

The relative expression of TSHR and NIS mRNA was evaluated using RT-PCR to test the effect of exogenous progestin on the transcription of target genes involved in the HPT axis.

**Figure 2:**
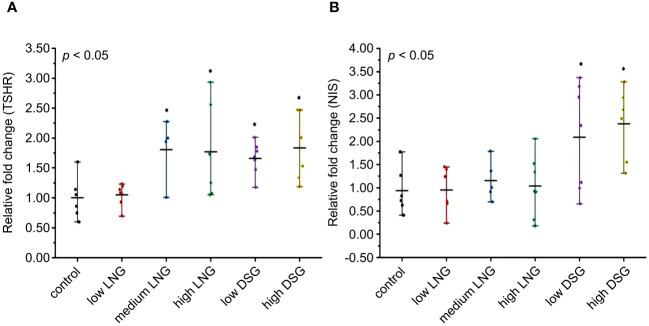
Effects of progestin on **(A)** TSHR and **(B)** NIS mRNA relative expression in rats. TSHR, thyroid stimulating hormone receptor; NIS, sodium iodide symporter; LNG, levonorgestrel, DSG, desogestrel. *p <0.05 was considered statistically significant, compared with the control group.

After the 30-day progestin gavage, the relative mRNA expression of TSHR differed significantly between the control and experimental groups (*p*=0.018) (**Figure 2A**). Progestin exposure resulted in a significant TSHR upregulation (1.81-fold in the medium LNG, 1.77-fold in the high LNG, 1.66-fold in the low DSG, and 1.93-fold in the high DSG groups).

Similarly, after the 30-day progestin gavage, the relative mRNA expression of NIS differed significantly between the control and experimental groups (*p*=0.004) (**Figure 2B**). After *post-hoc* examination, DSG exposure resulted in a significant upregulation of NIS expression (2.09-fold in the low DSG group and 2.38-fold in the high DSG group).

### 3.5 THRα and THRβ expression in tissues following progestin exposure

**Figure 3:**
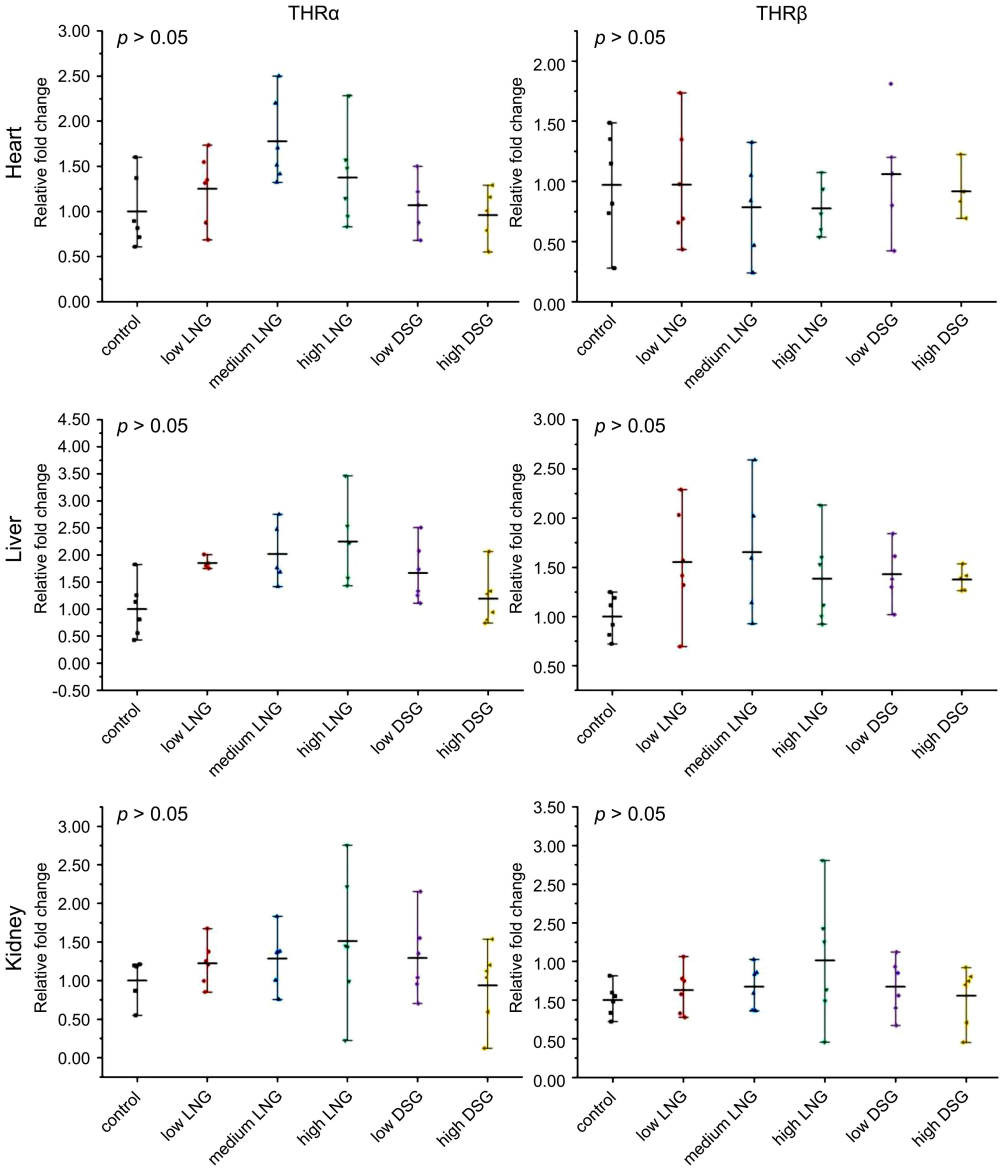
Effects of progestin on THRα and THRβ mRNA relative expression in the heart, liver, and kidney of the rats in the groups. THRα, thyroid hormone receptor α; THRβ, thyroid hormone receptor β; LNG, levonorgestrel, DSG, desogestrel.

The relative expression of THRα and THRβ mRNA was evaluated using RT-PCR to test the effects of exogenous progestin on THR expression. The results are shown in **Figure 3**.

In the heart, the relative expression of THRα mRNA in low, medium and high LNG groups and low and high DSG groups was not statistically significant compared with the control group respectively; the relative mRNA expression of THRβ was not statistically significant compared with the control group.

In the liver, the relative expression of THRα mRNA in the low, medium and high LNG groups and low and high DSG groups was not statistically significant compared with the control group respectively; the relative mRNA expression of THRβ was not statistically significant compared with the control group.

In the kidney, compared with the control group, the relative mRNA expression of THRα in the low, medium, and high LNG, and the DSG groups was not statistically significant compared with the control group, also that of the relative mRNA expression of THRβ.Overall, exogenous progestin increased the gene expression of THRα and THRβ in the kidneys; however, this difference was not statistically significant.

### 3.6 Changes in thyroid histomorphology

**Figure 4:**
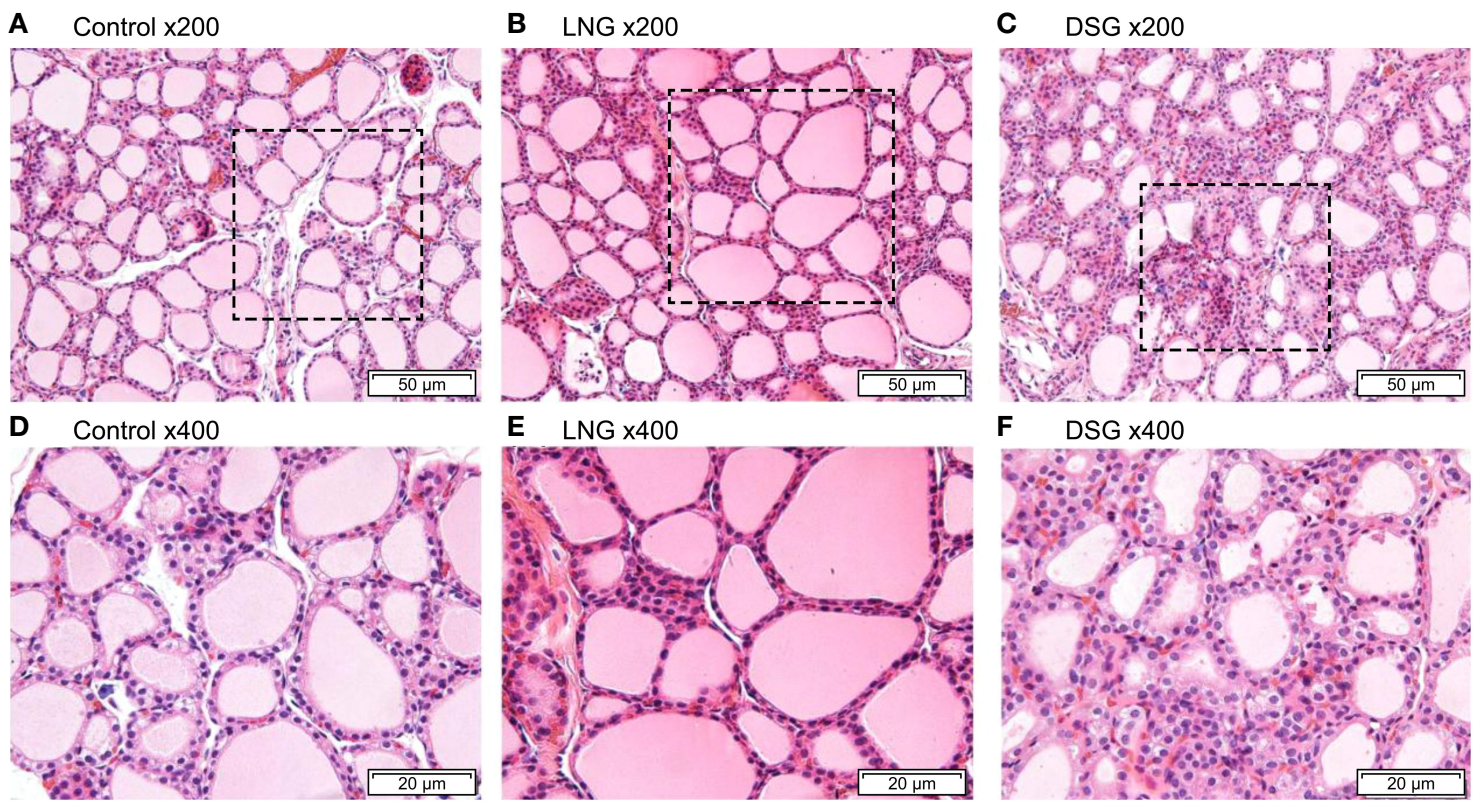
Hematoxylin and eosin staining of rat thyroid tissues. **(A)** control group (magnification, x200); **(B)** LNG group (magnification, x200); **(C)** DSG group (magnification, x200); **(D)** control group (magnification, x400); **(E)** LNG group (magnification, x400); **(F)** DSG group (magnification, x400). LNG, levonorgestrel, DSG, desogestrel.

The H&E-stained images of the thyroid tissue are shown in **Figure 4**. The thyroid follicles of the rats in the control group were intact and evenly distributed, and the thyroid follicular epithelial cells were neatly arranged, with uniformly sized follicles, round or oval nuclei, and abundant colloid in the follicular lumen (**Figures 4A, D**). After progestin stimulation, the thyroid tissue became pleomorphic, with follicles of varying sizes. Three thyroid samples were selected from each group, and three visual fields were taken from each thyroid sample to quantitatively observe thyroid follicular cells. As presented in **Supplementary Table S2**, compared with the control group, the number of thyroid follicles in the experimental group increased. In the LNG group, the main manifestation was the varying size of the thyroid follicles (**Figures 4B, E**), while follicular epithelial cell hyperplasia was evident in the DSG group (**Figures 4C, F**).

## 4 Discussion

Our study offers novel insights for clinical research into thyroid disorders in childbearing women. This study was conducted on intact rats without gonadectomy, and exogenous progestin was administered to address the lack of focus on exogenous progestin in previous studies (4). Thus confirming that chronic exposure to exogenous progestin may cause thyroid dysfunction, reduce serum thyroid hormone antibody levels, upregulate TSHR and NIS transcription in the HPT axis, and stimulate thyroid follicular epithelial proliferation in rats.

The adverse effects of OCs, in addition to their contraceptive effects, have been reported with their expanded and prolonged use. The common side effects of OCs are breast pain, headache, tension, vomiting, acne, fatigue, decreased libido, and elevated blood pressure (27). Some women gain weight after taking the pill due to certain ingredients. Elevated estrogen levels during the first 3 months of OC use cause water and sodium retention, leading to weight gain during the second half of the menstrual cycle. Conversely, progestin promotes anabolism, which can also lead to weight gain. However, female juvenile rats fed approximately 80-fold the adult amount of long-acting norgestrel for 3 consecutive days showed significantly lower body weight gain than did the control group (28), contrary to results from human studies. We observed no significant difference in the body weight of the sexually mature female rats between the experimental and control groups in our study, consistent with the results of another study in which 6–7 week-old KM mice were treated with LNG (0.1 mg/d) for 5 days (29). These results indicate that progesterone did not cause significant changes in body weight.

Progesterone is primarily secreted by the ovaries in non-pregnant females and plays a crucial role in pregnancy maintenance. The low serum progesterone levels in this study indicated that exogenous progestin could directly inhibit progesterone secretion by inhibiting the ovary. Progesterone directly suppresses estrogen and progesterone production by ovarian granulosa cells (30, 31). Acting on the hypothalamic-pituitary-gonadal axis, it exerts a strong anti-gonadotropic effect in the hypothalamus, inhibiting ovulation, thickening the uterus, reducing uterine mucus, and inhibiting progesterone secretion. Our findings are consistent with the results of a study on gerbils with a single LNG exposure and SD rats receiving olive oil (0.2 ml/100 g/day) containing a combination of 1.0 μg ethinylestradiol and 10.0 μg levonorgestrel (30, 31). A small randomized controlled trial reported significantly reduced TSH levels in a 12-week trial of oral trace progesterone, 300 mg/d at bedtime, in postmenopausal women (32), which is consistent with our study. In animal studies, progestin administered intramuscularly for 30 days (1 mg/100 g/d) had minimal appreciable effects on serum TSH levels in female rats with intact gonads (33), which is similar to the results of serum TSH levels under DSG exposure in our study. In this study, after 30-day LNG gavage, the serum FT_4_ and TSH levels were lower in the experimental group than that in the control group. After DSG gavage, the serum FT_3_ and FT_4_ levels in the experimental group were lower than those in the control group; however, the serum TSH levels did not differ significantly. A previous study reported that contraceptives induced increased thyroxine-binding globulin (TBG) synthesis in the liver (34), which may explain the discrepancy in the results. The direct effect of elevated serum TBG concentration may enhance thyroxine binding, thereby reducing FT_4_ levels. Therefore, the low FT_4_ level in this study may be influenced by TBG. The decrease in the serum FT_4_ and FT_3_ levels is an important serological indicator of hypothyroidism; however, it is often accompanied by an increase in the serum TSH level. Hypothyroidism is a disease that results in reduced metabolism in the body because of decreased TH synthesis and secretion, or their physiological effects. In this study, the serum THs and TSH levels were lower in the experimental group than that in the control group, possibly because of progestin stimulation, which caused abnormalities in the thyroid pituitary axis. In clinical practice, this condition is called central hypothyroidism, wherein measuring other hormones released by the pituitary gland is required for further clarification. A review suggested that contraceptive use is associated with a high risk of hypothyroidism, and women taking contraceptives for >10 years have a higher susceptibility to hypothyroidism (35), which is consistent with our results. In addition, studies on rats showed that the long-term use of estrogen and progesterone increases the incidence of pituitary tumors (36), suggesting the effects of estrogen and progestin on the pituitary gland. Similar to LNG, serum TH levels were lower than that in the control group in rats exposed to low DSG. However, the TH levels were higher than that in controls at high DSG exposure, which may be attributed to the difference between LNG and DSG. Compared with LNG, users of contraceptives containing DSG had higher sex hormone-binding globulin (SHBG) levels (37), and SHBG levels were positively correlated with TH levels (38). Therefore, the effect of serum TBG on THs may be masked by the effect of SHBG, given that the increase in serum TBG level was less pronounced than that of SHBG (39). TH antibodies are autoantibodies produced by the immune system against components of the thyroid gland itself. The level of antibodies and the iodine content of the body are related. High antibody levels tend to be associated with higher levels of iodine in the body, and antibody levels decrease when iodine levels in the body are decreased. Therefore, in this study, the decrease in TGAb and TPOAb may be due to progesterone enhancing iodine excretion, which reduces the retention of iodine in the body, thus affecting antibody levels.

The present study demonstrated that after progestin exposure, the serum FT_3_ and FT_4_ levels were lower in the experimental group than in the control group, and that progestin regulated some HPT-axis-related genes in rats. The transcription of the TSH gene can serve as a toxicological endpoint for evaluating the mechanism of action of chemicals in the thyroid system. As a plasma membrane glycoprotein, NIS mediates the transport of active iodine ions to thyroid follicular cells, which is also a key first step in TH synthesis (40). In this study, the exogenous progesterone increased the expression of TSHR mRNA to compensate for reduced thyroid hormone levels, thereby inhibiting growth. Therefore, the upregulation of NIS mRNA expression may be a compensatory mechanism for the reduction in TH; a similar result was found in adult zebrafish (41). Additionally, THs interact with nuclear receptors (THRα and THRβ), trigger specific physiological effects, and play an important role in rat development. Therefore, the transcriptional expression levels of the THRα and THRβ genes were determined as indicators for circulating TH levels. The expression of THR isoforms is tissue-dependent and developmentally regulated (42). The expression levels of THR isoforms are diverse in different tissues; for example, the major THR isoform in the heart is THRα, and that in the kidney is THRβ. We reported slight alterations in the mRNA expression of THRα and THRβ following exposure to progestin, demonstrating no significant effects on the expression of THRα and THRβ mRNA in response to progestin exposure.

The most basic functional unit of the thyroid gland is the thyroid follicular epithelium. In this study, pathological changes, such as thyroid follicular epithelial cell hyperplasia, were detected in the thyroid gland. Rats in the experimental group had smaller follicular cavities and different sizes of follicles than did those in the control group. This hyperplasia of thyroid follicles may have been attributed to insufficient synthesis of THs in the body.

However, a limitation of this study was its focus mainly on changes in serum TH levels, and lacks the exploration and analysis of its potential mechanism, and does not clarify the mechanism of its occurrence. Future research is needed to further understand the underlying molecular mechanisms by which progesterone affects thyroid function, as well as the disruptions in the thyroid endocrine system by other synthetic progestins with different chemical structures.

In conclusion, our results showed that progestin exposure may lead to thyroid dysfunction, reduced thyroid hormone antibody levels, and upregulation of the transcription of TSHR and NIS in the HPT axis, thus inducing patho-morphological changes in female Wistar rats. However, these changes were manageable and did not cause any significant thyroid disorders, such as goiters, thyroid nodules, or thyroiditis. Our results suggest that women, particularly those with thyroid disease, should be cautious when taking OCs, and that more studies focusing on the mechanism of OCs’ effect on thyroid function are warranted.

## Data availability statement

The raw data supporting the conclusions of this article will be made available by the authors, without undue reservation.

## Ethics statement

The animal study was approved by Ethics Committee of Harbin Medical University (approval number: hrbmu202108). The study was conducted in accordance with the local legislation and institutional requirements.

## Author contributions

HX: Writing – original draft. TQ: Data curation, Conceptualization, Writing – original draft. LL: Software, Investigation, Writing – review & editing. RS: Validation, Investigation, Writing – original draft. WC: Investigation, Formal analysis, Writing – review & editing. MZ: Resources, Investigation, Writing – review & editing. XH: Investigation, Data curation, Writing – review & editing. HP: Investigation, Writing – review & editing. YS: Supervision, Investigation, Writing – review & editing. JL: Visualization, Investigation, Writing – review & editing. XD: Writing – review & editing. PL: Conceptualization, Writing – review & editing.
