# Supplementary material for: Effect of progestin on thyroid function in female Wistar rats
Source: Front Endocrinol (Lausanne). 2024 Jun 5;15:1362774. doi: 10.3389/fendo.2024.1362774 (PMC11188309; doi:10.3389/fendo.2024.1362774)
Supplement: Supplementary file 1 [file DataSheet_1.pdf]

Supplementary Materials

Supplementary Table S1. Groups of rats and specific gavage dose

**Group**		**N**	**30-days**
control		20	0.5ml peanut oil
LNG	low LNG	20	0.0039mg*20 fold
	medium LNG	20	0.0039mg*100 fold
	high LNG	20	0.0318mg*100 fold
DSG	low DSG	20	0.0083mg*20 fold
	**high DSG**	**20**	**0.0083mg*100 fold**

LNG, levonorgestrel; DSG, desogestrel. The 20 and 100 were magnified fold. Progestin was dissolved in 0.5ml peanut oil to gavage every 4 days.

Supplementary Table S2. Histomorphometric analysis of thyroid gland sections of Wistar rats.

**Group**	**Number of Thyroid follicles**
control	596.44±58.8
low LNG	712.33±115.79
medium LNG	716.67±170.81
high LNG	702.67±175.29
low DSG	811.83±138.89*
high DSG	802.17±125.06*
P	0.041

Results were presented as $\bar{x}$±s for normal distribution data LNG, levonorgestrel; DSG, desogestrel; p< 0.05 was considered statistically significant. *p<0.05 vs. control.
